# Indication and benefit of upfront hematopoietic stem cell transplantation for T-cell lymphoblastic lymphoma in the era of ALL-type induction therapies

**DOI:** 10.1038/s41598-020-78334-x

**Published:** 2020-12-08

**Authors:** Mari Morita-Fujita, Yasuyuki Arai, Satoshi Yoshioka, Takayuki Ishikawa, Junya Kanda, Tadakazu Kondo, Takashi Akasaka, Yasunori Ueda, Kazunori Imada, Toshinori Moriguchi, Kazuhiro Yago, Toshiyuki Kitano, Akihito Yonezawa, Masaharu Nohgawa, Akifumi Takaori-Kondo, Kyoto Stem Cell Transplantation Group (KSCTG)

**Affiliations:** 1 Department of Hematology and Oncology, Graduate School of Medicine, Kyoto University, Kyoto, Japan; 2 Department of Hematology, Kobe City Medical Center General Hospital, Hyogo, Japan; 3 Department of Clinical Laboratory Medicine, Graduate School of Medicine, Kyoto University, Kyoto, Japan; 4 Department of Hematology, Tenri Hospital, Nara, Japan; 5 Department of Hematology, Kurashiki Central Hospital, Okayama, Japan; 6 Department of Hematology, Japanese Red Cross Osaka Hospital, Osaka, Japan; 7 Department of Hematology, Kyoto-Katsura Hospital, Kyoto, Japan; 8 Department of Hematology, Shizuoka General Hospital, Shizuoka, Japan; 9 Department of Hematology, Kitano Hospital, Osaka, Japan; 10 Department of Hematology, Kokura Memorial Hospital, Fukuoka, Japan; 11 Department of Hematology, Japanese Red Cross Wakayama Medical Center, Wakayama, Japan

**Keywords:** Epidemiology, Outcomes research

## Abstract

Since the introduction of leukemia-type induction therapies for T-cell lymphoblastic lymphoma (T-LBL), improvements in the long-term outcomes of T-LBL have been reported. However, indications for and the appropriate timing of hematopoietic stem cell transplantation (HSCT) have not yet been established. Therefore, we performed a multicenter retrospective cohort study of patients with T-LBL treated using leukemia-type initial therapies to compare the outcomes after HSCT at different disease stages. We enrolled 21 patients with T-LBL from a total of 11 centers, and all patients received hyper-CVAD as a leukemia-type initial regimen. HSCT was performed during the CR1/PR1 (standard disease) stage in 11 patients, while it was completed at a later or non-remission (advanced disease) stage in 10 patients. Following HSCT, the overall survival rate was significantly greater in standard disease than in advanced-disease patients (79.5% vs. 30.0% at 5 years; hazard ratio (HR) 5.97; *p* = 0.03), with trend to the lower incidence of relapse in the former group (27.3% vs. 60.0% at 5 years; HR 2.29; *p* = 0.19). A prognostic difference was not detected between cases treated with allogeneic and autologous HSCTs. Our study suggests that frontline HSCT may be a feasible treatment option for T-LBL, even in the era of leukemia-type initial therapy.

## Introduction

T-cell lymphoblastic lymphoma (T-LBL) is a rare hematological malignancy that is characterized by mediastinal lesions with minimal bone marrow infiltration^1–3^. Despite its known cellular origin in lymphoblasts, T-LBL is distinguished from acute lymphoblastic leukemia (ALL) due to its distinct clinical presentation involving predominant mass lesions and infrequent bone marrow infiltration (blasts < 20%)^4^, instead demonstrating clinical features more similar to those of non-Hodgkin’s lymphoma (NHL). Therefore, NHL-type chemotherapeutic regimens have previously been chosen as initial therapies in patients with T-LBL, but unlike in normal NHL cases, upfront hematopoietic stem cell transplantation (HSCT) was also often recommended in order to suppress late-phase relapse and promote more favorable outcomes^5,6^.

Recently, ALL-type regimens including hyper CVAD (composed of fractionated cyclophosphamide, vincristine, doxorubicin, and dexamethasone) and high-dose cytarabine combined with methotrexate have been standardized as the new initial therapies for T-LBL based on studies reporting higher remission and lower relapse rates in the early phase^7^. However, under this novel ALL-type initial therapy approach, the indication and appropriate timing of HSCT, if necessary, remain unestablished because the validity of the risk stratification system for relapse after chemotherapies is not confirmed^2,8^, and moreover, the beneficial effects of HSCT have not yet been evaluated according to each disease status of T-LBL^9–11^.

Therefore, we performed the present multicenter retrospective cohort study of patients with T-LBL treated with ALL-type initial therapies to (1) determine the risk factors for relapse after ALL-type chemotherapies in patients with T-LBL, and (2) compare the outcomes after HSCT performed at different time points and in patients with different disease statuses. Further, this study may provide information helpful in determining the optimal treatment approach to use to attain more favorable outcomes among patients with T-LBL in the current ALL-type therapy era.

## Subjects and methods

### Inclusion criteria

Data on adult patients (age ≥ 16 years) who underwent their first HSCT between January 2000 and September 2016 at hospitals of the Kyoto Stem Cell Transplantation Group (KSCTG) were obtained through the Japanese Transplant Registry Unified Management Program (TRUMP), which is sponsored by the Japanese Society for Hematopoietic Cell Transplantation and Japanese Data Center for Hematopoietic Cell Transplantation^12^. We included HSCT-eligible patients who fulfilled the following World Health Organization 2016 criteria for T-LBL^4^. The study protocol complied with the standards outlined in the Declaration of Helsinki and was approved by the ethical committee of Kyoto University (R-1507). Written informed consent was obtained from each patient or from a parent and/or legal guardian in case of patients below 18 years of age.

### Data collection and definition of each covariate

We extracted data from the KSCTG database on basic pretransplant characteristics and the posttransplant clinical course. We also reviewed the medical records and extracted the data available there and discerned the HSCT characteristics and posttransplant clinical course details according to the predefined standardized protocol. The clinical stage was determined according to the Ann Arbor system, with the initial evaluation of bone marrow for all the patients being mandatory. The overall disease risk was calculated using the International Prognostic Index (IPI) at diagnosis. Treatment responses, including relapse, were evaluated according to the Cheson criteria^13^. The disease status at the time of HSCT was categorized as standard and advanced disease; the prior encompassed patients transplanted at CR1 or PR1 status, while the latter included those transplanted at CR2/PR2 or later, primary induction failure (PIF), or stable (SD) or progressive disease (PD) after the consequences of any chemotherapy. Regarding conditioning regimens, myeloablative conditioning (MAC) and reduced-intensity conditioning (RIC) were defined based on the previously published consensus criteria^14^.

### Statistical analyses

Patient characteristics were compared between the standard- and advanced-disease groups using Fisher’s exact tests for categorical variables and the Mann–Whitney U test for continuous variables. For survival analysis, the overall survival (OS) was measured from the date of diagnosis or HSCT to the last follow-up visit; survival curves were described using the Kaplan–Meier method, and the groups were compared using the log-rank test. Cumulative incidence curves for non-relapse mortality (NRM) and relapse were compared using the Gray test, treating relapse and NRM as competing risks, respectively. For the statistical analysis of these prognostic factors, we employed Cox proportional-hazards regression model and the Fine–Gray proportional-hazards models^15^. Multivariate analyses included those variables showing the significance (or clinically relevant) in the preceding univariate analyses, and the number of variables in the model was restricted to one per 5–10 events. All statistical analyses were performed using the R statistical software program, version 3.6.1 (R Foundation for Statistical Computing, Vienna, Austria). All p-values are two-sided, and *p* < 0.05 was considered to be statistically significant.

## Results

### Patient characteristics

From the total cohort of 2,425 patients, we chose 82 patients categorized as precursor lymphoid neoplasms, finally enrolling a total of 21 patients with T-LBL who received HSCT in a total of 11 centers. The median age at the time of diagnosis was 34 (range 17–54) years (Table 1). The clinical stage at diagnosis ranged from III to IV in 18 patients (85.7%); 13 patients (61.9%) possessed mediastinal lesions, while bone marrow involvement was confirmed in 9 patients (42.9%). The performance status (PS) was 1 or less in 13 patients (61.9%). Meanwhile, the IPI score was 3 or greater (high risk) in 10 patients (47.6%). Other notable patient characteristics at diagnosis are included in Table 1.

**Table 1:** Patient characteristics at the time of diagnosis.

Variables		*N* = 21
Age, y	Median (Range)	34 (17–54)
	< 35/ ≥ 35	12 (57.1%)/9 (42.9%)
Sex	Female/Male	7 (33.3%)/14 (66.7%)
PS	0–1/2–4	13 (61.9%)/8 (38.1%)
Ann Arbor stage	I-II/III-IV	3 (14.3%)/18 (85.7%)
IPI	0–2/3–5/NA	10 (47.6%)/10 (47.6%)/1 (4.8%)
BM involvement	Y (blast%; median, range)	9 (42.9%), 3.2% (1.8%-18.5%)
Extranodal lesions	Mediastinum/Pleura/Pericardium	13 (61.9%)/11 (52.4%)
	Lung/Liver/GI tract	3 (14.3%)/2 (9.5%)/2 (9.5%)
	CNS	0 (0.0%)
LDH	≤ / > ULN/NA	12 (57.1%)/8 (38.1%)/1 (4.8%)

*PS* performance status, *IPI* international prognostic index, *NA* not available, *BM* bone marrow, *GI* gastrointestinal, *CNS* central nervous system, *LDH* lactate dehydrogenase, *ULN* upper limit of normal.

### Pre-transplant initial treatments

**Figure 1:**
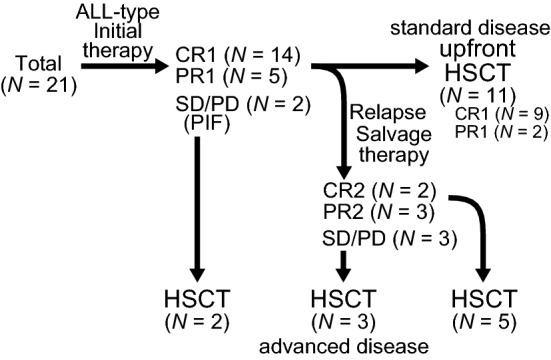
Schematic workflow of patients and the pre-HSCT clinical course. Summary of treatment response from diagnosis to HSCT. Upfront HSCT at CR1/PR1 was categorized as standard disease, while HSCT performed in the other status was classified as advanced disease.

The pretransplant clinical course is summarized in Fig. 1. All patients received ALL-type initial regimens composed of hyper-CVAD or its derivatives [i.e., the addition of cytarabine, 6-mercaptopurine, and/or L-asparaginase (L-ASP)] with or without high-dose methotrexate and/or cytarabine-containing regimens. Prior to introducing the ALL-type initial regimens, four patients received one or two courses of NHL-type regimens including CHOP (cyclophosphamide, vincristine, doxorubicin, and prednisolone) or ICE (ifosfamide, carboplatin, etoposide)-like regimens. The details of initial therapies (induction and consolidation regimens) are summarized in Supplemental Table 1.

After the abovementioned initial treatments were given, 19 patients achieved their first CR (CR1; *N* = 14; 66.7%) or first PR (PR1; *N* = 5; 23.8%) (Fig. 1). Those who failed to achieve CR/PR in the initial therapies underwent HSCT at the stage of SD/PD (*N* = 2). Among CR1/PR1 patients, 11 patients underwent upfront HSCT, while other 8 patients did not. All of the 8 patients experienced relapse, and salvage therapies were introduced, including monotherapies (nelarabine, clofarabine, and L-ASP) and the combination of mitoxantrone, etoposide, medium-dose cytarabine. Five patients responded to the salvage therapies and underwent HSCT at second CR (CR2; *N* = 2) or second PR (PR2; *N* = 3) (Fig. 1). Those who failed to achieve CR2/PR2 with salvage therapy were transplanted at SD/PD disease status.

Refractoriness and/or relapse before HSCT was observed in 10 patients during the course of or after the initial chemotherapies; 2 patients did not achieve remission after the induction therapies (PIF) and 8 patients experienced relapse after initial therapies (Fig. 1). Risk analyses using the Fine–Gray proportional-hazards model demonstrated that none of the patient characteristics at diagnosis were significantly correlated with the occurrence of relapse (data not shown). These results support that the risk of relapse cannot be predicted at the time the diagnosis is made, and all patients should be regarded as being at high risk for relapse once diagnosed with T-LBL.

### HSCT procedures and post-transplant outcomes

Patient characteristics at the time of HSCT are shown in Table 2. The disease status profile of the study group at the time of HSCT is as follows: CR1 (*N* = 9), PR1 (*N* = 2), CR2 (*N* = 2), PR2 (*N* = 3), SD/PD (*N* = 3), and PIF (*N* = 2). Thus, 11 patients were categorized as standard-disease patients, and 10 patients were categorized as advanced-disease patients. The median time from diagnosis to HSCT in the total study group was 8.2 (2.3–20.4) months; the median time was 6.9 (5.0–9.6) months in the standard-disease group, while it was 10.7 (2.3–20.4) months in the advanced-disease group. Allogeneic donors were selected in 16 cases (76.2%; bone marrow or peripheral blood stem cell transplantation, *N* = 11, and cord blood transplantation, *N* = 5), while 5 patients (23.8%) underwent autologous HSCT because allogeneic donors were not available in a timely manner. MAC regimens were adopted in 16 patients (76.2%), while 5 patients (23.8%) received RIC. Detailed information on conditioning regimens is presented in the Supplemental Table 2. No significantly skewed distributions of patient characteristics or HSCT parameters between the standard- and advanced-disease groups were observed (Table 2).

**Table 2:** Patient characteristics related to HSCT.

Variables		Standard-disease (*N* = 11)	Advanced-disease (*N* = 10)	*p*
Age at HSCT, y	< 35/ ≥ 35	5 (45.5%)/6 (54.5%)	6 (60.0%)/4 (40.0%)	0.67
Sex	Female/Male	3 (27.3%)/8(72.7%)	4 (40.0%)/6 (60.0%)	0.66
PS	0–1/2–4	11 (100%)/0 (0.0%)	9 (90.0%)/1 (10.0%)	0.48
HCT-CI	0/1-	9 (81.8%)/2 (18.2%)	7 (70.0%)/3 (30.0%)	0.64
Disease status	CR/PR	9 (81.8%)/2 (18.2%)	2 (20.0%)/3 (30.0%)	0.01*
(response to chemo)	SD/PD	NA	5 (50.0%)	
Periods from Dx to HSCT, d	Median (range)	209 (152–291)	325 (71–621)	0.11
Donor source	Auto/Allo	3 (27.3%)/8 (72.7%)	2 (20.0%)/8 (80.0%)	1.00
	(allo) BM/PB/CB	7 (87.5%)/1 (12.5%)	4 (50.0%)/4 (50.0%)	0.28
Conditioning regimen	(auto) MAC/RIC	2 (66.7%)/1(33.3%)	0 (0.0%)/2 (100%)	0.40
	(allo) MAC/RIC	8 (100%)/0 (0.0%)	6 (75.0%)/2 (25.0%)	0.47
GVHD prophylaxis (allo)	CNI + MTX / + MMF	7 (87.5%)/1(12.5%)	3 (37.5%)/4 (50.0%)	0.12
Year of HSCT	2000–2009/2010–2016	5 (45.5%)/6 (54.5%)	3 (30.0%)/7 (70.0%)	0.66
Follow-up for survivors, y	Median (range)	9.3 (3.2–16.2)	8.0 (4.8–10.0)	0.60

*HSCT* hematopoietic stem cell transplantation, *HCT-CI* hematopoietic cell transplantation-specific comorbidity index, *CR* complete remission, *PR* partial remission, *SD* stable disease, *PD* progressive disease, *Dx* diagnosis, *Auto* autologous, *Allo* allogeneic, *PB* peripheral blood, *CB* cord blood, *MAC* myeloablative conditioning, *RIC* reduced intensity conditioning, *GVHD* graft-versus-host disease, *CNI* calcineurin inhibitor, *MTX* methotrexate, *MMF* mycophenolate mofetil. Other abbreviations are shown in Table 1.

*Statistically significant.

**Figure 2:**
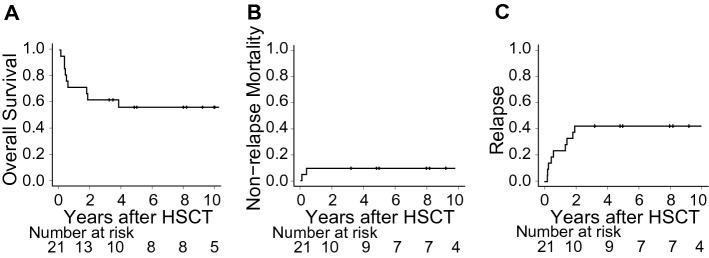
Prognosis after HSCT in the whole cohort. (**A**) Overall survival after HSCT was calculated using the Kaplan–Meier method. (**B**) Non-relapse mortality is shown treating relapse as a competing risk. (**C**) The cumulative incidence of relapse was calculated treating death without relapse as a competing risk.

After the median follow-up time of 8.8 (3.2–16.2) years for survivors, post-HSCT relapse was observed in 9 patients, while NRM was marked in 2 patients. Figure 2 shows the curves for OS (at 5 years from HSCT, 56.3%; 95% CI 32.6–74.5%), NRM (at 5 years, 9.5%; 95% CI 1.5–26.7%), and the cumulative incidence of relapse (at 5 years, 42.9%; 95% CI 21.2–63.0%) for the entire cohort.

### HSCT-related prognostic factors

**Figure 3:**
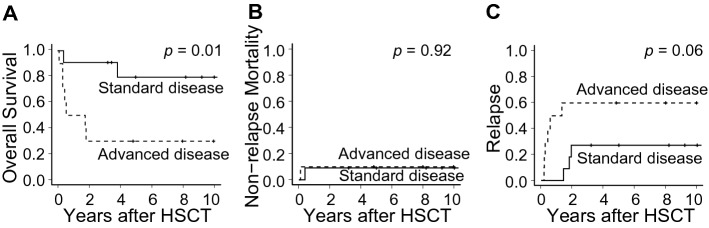
Comparison of post-HSCT prognosis according to the disease risk at HSCT. Comparison of prognosis between standard disease (HSCT at CR1/PR1) and advanced disease (HSCT at CR2/PR2 or at non-remission status) regarding (**A**) overall survival, (**B**) non-relapse mortality, and (**C**) relapse.

Next, aspects of the posttransplant prognosis including, OS, NRM, and relapse, were compared between standard- and advanced-disease patients to determine the appropriate timing of HSCT among the available strategies for T-LBL. As a result, OS from HSCT was superior in the standard-disease group relative to the advanced-disease group at five years (79.5%; 95% CI 39.3–94.5% vs. 30.0% (95%CI 7.1–57.8%), *p* = 0.01) (Fig. 3A). NRM was in the similar trend (Fig. 3B), while standard-disease patients showed lower relapse with borderline significance when compared with advanced-disease patients (60.0%; 95% CI 21.7–84.3% vs. 27.3%; 95% CI 5.7–55.4%; *p* = 0.06) (Fig. 3C).

The abovementioned prognostic differences between the two groups concerning the disease risk were subjected to the adjustment by the other confounding factors; univariate and multivariate analyses incorporating various prognostic factors were performed, and the results are shown in Table 3. In the univariate analysis for OS, HCT-CI ≥ 1 was associated with the inferior OS as compared with HCT-CI 0 with the borderline significance [hazard ratio (HR), 3.43; 95% CI 0.91–12.9; *p* = 0.07], while the advanced-disease patients presented an HR of 5.92 (95% CI 1.22–28.8; *p* = 0.03) as compared with the standard-disease patients. The other considered factors were not statistically significant including the donor origin (allogeneic vs. autologous), and multivariate analysis indicated that disease risk remained the significant risk factor for poorer OS after HSCT (HR 5.97; 95% CI 1.21–29.4; *p* = 0.03) (Table 3).

**Table 3:** Univariate and multivariate analyses of prognostic factors.

Variables		Overall survival				Relapse			
		Univariate		Multivariate		Univariate		Multivariate	
		HR (95%CI)	*p*	HR (95%CI)	*p*	HR (95%CI)	*p*	HR (95%CI)	*p*
**Factors at diagnosis**									
Sex	Male	1.00 (*reference*)				1.00 (*reference*)			
	Female	1.16 (0.29–4.66)	0.83			1.29 (0.30–5.46)	0.73		
PS	0–1	1.00 (*reference*)				1.00 (*reference*)			
	2–4	0.79 (0.20–3.16)	0.74			1.42 (0.40–4.97)	0.59		
Ann Arbor stage	I–II	1.00 (*reference*)				1.00 (*reference*)			
	III–IV	0.61 (0.13–2.98)	0.55			0.47 (0.11–2.01)	0.31		
IPI	0–2	1.00 (*reference*)				1.00 (*reference*)			
	3–5	0.56 (0.13–2.33)	0.42			1.77 (0.44–7.04)	0.42		
BM involvement	N	1.00 (*reference*)				1.00 (*reference*)		1.00 (*reference*)	
	Y	0.61 (0.15–2.44)	0.48			0.25 (0.06–1.03)	0.06	0.35 (0.06–1.93)	0.23
Mediastinal lesions	N	1.00 (*reference*)				1.00 (*reference*)			
	Y	0.65 (0.17–2.43)	0.52			1.06 (0.26–4.38)	0.94		
LDH	≤ ULN	1.00 (*reference*)				1.00 (*reference*)			
	> ULN	0.49 (0.10–2.41)	0.38			0.92 (0.23–3.67)	0.91		
**Factors at HSCT**									
Age at HSCT, y	< 35	1.00 (*reference*)				1.00 (*reference*)			
	≥ 35	0.79 (0.21–2.97)	0.73			0.95 (0.27–3.37)	0.93		
HCT-CI	0	1.00 (*reference*)		1.00 (*reference*)		1.00 (*reference*)		1.00 (*reference*)	
	1-	3.43 (0.91–12.9)	0.07	3.48 (0.89–13.65)	0.07	4.53 (1.24–16.5)	0.02*	4.80 (0.96–24.1)	0.06
Disease risk	Standard	1.00 (*reference*)		1.00 (*reference*)		1.00 (*reference*)		1.00 (*reference*)	
	Advanced	5.92 (1.22–28.8)	0.03*	5.97 (1.21–29.4)	0.03*	3.62 (1.02–12.9)	0.06	2.29 (0.66–7.93)	0.19
Donor source	Auto	1.00 (*reference*)				1.00 (*reference*)			
	Allo	1.37 (0.28–6.60)	0.70			0.65 (0.20–2.18)	0.49		
Conditioning regimen	MAC	1.00 (*reference*)				1.00 (*reference*)		1.00 (*reference*)	
	RIC	3.23 (0.85–12.2)	0.09			5.00 (1.26–19.6)	0.02*	1.76 (0.32–9.82)	0.52
Year of HSCT	-2009	1.00 (*reference*)				1.00 (*reference*)			
	2010-	6.11 (0.76–49.2)	0.09			3.20 (0.80–12.7)	0.10		

*HR* hazard ratio, *CI* confidence interval. Other abbreviations are shown in Tables 1 and 2.

The results concerning relapse are shown in Table 3, and HSCT in the standard-disease group was linked to a lower incidence of relapse, together with a similar rate of NRM (Fig. 3B), resulting in the significantly superior OS seen in this cohort (Table 3).

**Figure 4:**
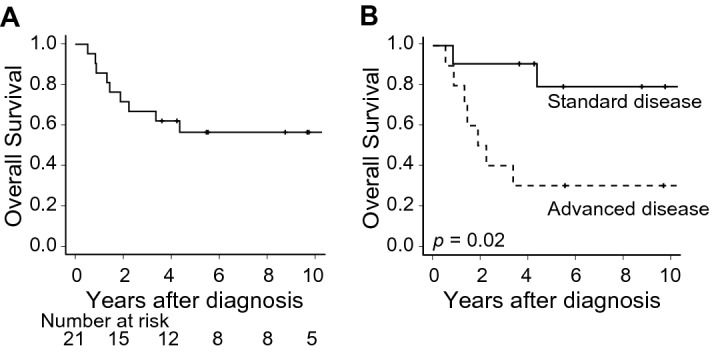
Overall survival after the initial diagnosis of T-LBL. (**A**) Overall survival after the time of initial diagnosis is shown, and (**B**) compared between standard- and advanced-disease patients.

Regarding donor source, our results indicated that post-HSCT outcomes were the same between patients who received autologous and allogeneic HSCT (Table 3), with the OS rates at 5 years being, 60.0% (95% CI 12.6–88.2%) and 54.7% (95% CI 27.4–75.5%) (*p* = 0.70), respectively (Supplemental Fig. 1A). NRM and incidence of relapse were also similar between these two subgroups (Supplemental Figs. 1B and 1C). The superiority regarding OS following HSCT in the standard-disease group was also confirmed by the analyses assessing OS from the initial diagnosis; the postdiagnosis OS in the entire cohort was 56.3% (95% CI 32.6–74.5%) (Fig. 4A) and was significantly better in the standard-disease group than in the advanced-disease group (79.5%; 95% CI 39.3–94.5% vs. 30.0%; 95% CI 7.1–57.8%; *p* = 0.02) (Fig. 4B), though a lead-time bias exists, negatively impacting standard-disease patients.

## Discussion

This multicenter retrospective cohort study investigated the risk of relapse and outcomes of HSCT in adult patients with T-LBL who received ALL-type initial therapies and revealed two major findings. (1) relapse after ALL-type chemotherapies without HSCT was frequently observed, and among considered patient- or disease-related variables, no significant risk factors for relapse were statistically extracted; (2) HSCT performed in patients with a CR1/PR1 status is associated with significantly more favorable outcomes (*i.e.*, improved OS both after HSCT and after the initial diagnosis) as compared with those experienced by patients with other disease statuses mainly as a result of the lower incidence of post-HSCT relapse. Our study highlights the potential beneficial role of frontline HSCT in adult patients with T-LBL, even in the era of ALL-type initial chemotherapy. The outlined considerations were explored for the first time in this study, though the size of the study cohort was small due to the rarity of this disease in adults.

Initially, the risk stratification of relapse after ALL-type chemotherapy is one of the most important aspects in T-LBL for the determination of HSCT indication. In our analyses, however, we failed to detect any patient group showing a significantly higher risk of relapse and who especially might require frontline HSCT among the patient subgroups we considered. These results, in part, can be attributed to a beta error^16^, but at the same time, may indicate the level of difficulty inherent in establishing T-LBL relapse risk-stratification models using the clinical parameters that are currently available. Recent studies have revealed that persistent minimal residual disease in bone marrow^17^, the mutation status of NOTCH1 and its downstream cascade^2^, and the expression of several microRNAs^8^ can possibly predict poorer prognosis in adult patients with T-LBL, but these biomarkers are not readily available in clinical practice, and their usefulness still need to be validated through prospective clinical trials. Until that time, all patients with T-LBL can be designated as candidates for HSCT because of the relatively high incidence of relapse after chemotherapy (from 35% up to 60%)^2,7,18,19^. Besides, using pediatric ALL-like chemotherapy (which is typically more intensified as compared with the ordinal ALL-like regimen) in young adult patients with T-LBL can suppress the relapse incidence more powerfully after chemotherapy, and this can limit the indication of HSCT^2^, although this was not analyzed in this study.

Now that certain records of the incidence rate of post-chemotherapy relapse exist and no significant risk factors have been nominated, HSCT should be considered in all eligible patients with T-LBL, and the timing of the transplant procedure should instead be the matter of debate. Regarding this point, we demonstrated that upfront HSCT (at CR1/PR1) can provide significantly superior prognosis when compared to that performed in patients with a CR2/PR2 or later state mainly due to the lower incidence of relapse. This difference in outcomes after HSCT performed at different times is significant not only in the comparison of the survival time after HSCT but also in that after the initial diagnosis, where the lead-time bias is unfavorable for upfront HSCTs. Considering the higher percentage of CR/PR patients in the upfront-HSCT cohort, the prognostic difference is mainly attributed to the pretransplant disease status and following relapse risk; patients with T-LBL, once having relapsed after chemotherapy, often experience difficulty in achieving CR/PR again prior to HSCT^20^, leading to the poor OS even after allo-HSCT^21^. In contrast with other hematological malignancies such as B-ALL, few promising therapeutic agents are currently available for relapsed T-LBL; nelarabine and clofarabine, which are candidates for T-LBL salvage therapy approved by the United States Food and Drug Administration, have shown only limited effects so far^22,23^. Considering that such salvage chemotherapies cannot guarantee the achievement of remission, at present, frontline HSCT may be the optimal feasible option for transplant-eligible patients.

The main suggestion taken away from our study–that is, to perform upfront HSCT in T-LBL initially treated with ALL-type chemotherapies is compatible with the findings of several other studies. In a multicenter retrospective study of 49 patients with LBL treated with the hyper-CVAD regimen, transplanted patients (*N* = 24) showed better OS (76% at 3 years) and progression-free survival (78% at 3 years) when compared with complete responders without HSCT consolidation^5^. Other studies have reported the superiority of HSCT consolidation after chemotherapies, but the wide variety of initial chemotherapies (ALL-type regimens were used only in half of the study group)^18^ and extremely shorter periods of observation (median was 31.5 months)^24^ unfortunately somewhat dilutes the external validity in these studies. On the other hand, one study suggested that allo-HSCT in CR1 should not be considered due to the relatively favorable outcomes even without HSCT^25^; their claim, however, depends on the prognosis in mature T-ALL/LBL patients, which is a relatively rare subtype in LBL^26^.

Regarding donor source (autologous vs. allogeneic), our analysis indicated the existence of a similar prognosis, but this evaluation is insufficient due to the small number of patients included, especially in the autologous HSCT cohort. Several studies have revealed a lower incidence of relapse in patients undergoing allogenic HSCT relative to autologous HSCT^8,11^; on the other hand, one study suggested the efficacy of tandem autologous HSCTs^27^. The results are inconclusive among all these studies due to the heterogeneity and the small number of cohorts, but it is suggested that allogenic HSCT is more effective from the viewpoint of perpetuating the continuous suppression of post-transplant relapse mainly due to the graft-versus-leukemia effects^11^. Autologous HSCT might be a treatment option in patients who are not eligible for allogenic HSCT.

Thus, the present study has analyzed the prognosis of HSCT-eligible patients with T-LBL, reviewing their clinical records comprehensively, yet some limitations to this study exist and must be addressed. First, the pretransplant therapy regimen (basically, hyper-CVAD–based regimens were adopted across the total cohort) and the timing or type of HSCT (at CR1/PR1 vs. later, allogeneic vs. autologous, or MAC vs. RIC regimens) were chosen by the physician in charge at that time, though, in most cases, allogeneic HSCT after the MAC regimen was selected. Second, the number of cases was small due to the rarity of the disease under study, and as a consequence, the evaluation of prognostic factors was insufficient. A prospective nationwide or international multicenter randomized trial in the future might overcome the limitations of this study.

## Conclusions

We performed a multicenter cohort retrospective study of patients with T-LBL, analyzing the efficacy of ALL-type regimens and the prognostic impacts of HSCT by obtaining all the necessary data from clinical records and evaluating these based on a standardized protocol. Our study indicated that frontline HSCT is a feasible treatment option in the era of intensive ALL-type therapy. We expect that this study may offer clinically useful information to improve the overall prognosis and trigger the identification of new therapeutic strategies for the patients with T-LBL in the future.

## Supplementary information

Supplementary Information.
