# Supplementary material for: Indication and benefit of upfront hematopoietic stem cell transplantation for T-cell lymphoblastic lymphoma in the era of ALL-type induction therapies
Source: Sci Rep. 2020 Dec 8;10:21418. doi: 10.1038/s41598-020-78334-x (PMC7722931; doi:10.1038/s41598-020-78334-x)
Supplement: Supplementary file 1 — Supplementary Information. [file 41598_2020_78334_MOESM1_ESM.pdf]

Supplemental Materials

Indication and benefit of upfront hematopoietic stem cell transplantation for T-cell lymphoblastic lymphoma in the era of ALL-type induction therapies

Short title: HSCT for T-cell lymphoblastic lymphoma

Mari Morita-Fujita[1,2], Yasuyuki Arai[1,3], Satoshi Yoshioka[2], Takayuki Ishikawa[2], Junya Kanda[1], Tadakazu Kondo[1], Takashi Akasaka[4], Yasunori Ueda[5], Kazunori Imada[6], Toshinori Moriguchi[7], Kazuhiro Yago[8], Toshiyuki Kitano[9], Akihito Yonezawa[10], Masaharu Nohgawa[11], and Akifumi Takaori-Kondo[1] on behalf of Kyoto Stem Cell Transplantation Group (KSCTG)

[1]Department of Hematology and Oncology, Graduate School of Medicine, Kyoto University, Kyoto, Japan;
[2]Department of Hematology, Kobe City Medical Center General Hospital, Hyogo, Japan;
[3]Department of Clinical Laboratory Medicine, Graduate School of Medicine, Kyoto University, Kyoto, Japan;
[4]Department of Hematology, Tenri Hospital, Nara, Japan;
[5]Department of Hematology, Kurashiki Central Hospital, Okayama, Japan;
[6]Department of Hematology, Japanese Red Cross Osaka Hospital, Osaka, Japan;
[7]Department of Hematology, Kyoto-Katsura Hospital, Kyoto, Japan;
[8]Department of Hematology, Shizuoka General Hospital, Shizuoka, Japan;
[9]Department of Hematology, Kitano Hospital, Osaka, Japan;
[10]Department of Hematology, Kokura Memorial Hospital, Fukuoka, Japan;
[11]Deparment of Hematology, Japanese Red Cross Wakayama Medical Center, Wakayama, Japan

Address for Correspondence: Yasuyuki Arai, M.D., Ph.D.
Department of Clinical Laboratory Medicine, and Hematology and Oncology, Graduate School of Medicine, Kyoto University
54, Shogoin Kawahara-cho, Sakyo-ku, Kyoto, 606-8507, Japan.
E-mail: ysykrai@kuhp.kyoto-u.ac.jp, Telephone: +81-75-751-4964, Fax: +81-75-751-4963

Supplemental Figure Legend

Supplemental Figure 1. Comparison of post-HSCT prognosis according to the donor source

Comparison of prognosis between auto- and allo-HSCT regarding (**A**) overall survival, (**B**) non-relapse mortality, and (**C**) relapse.

Supplemental Figure 1

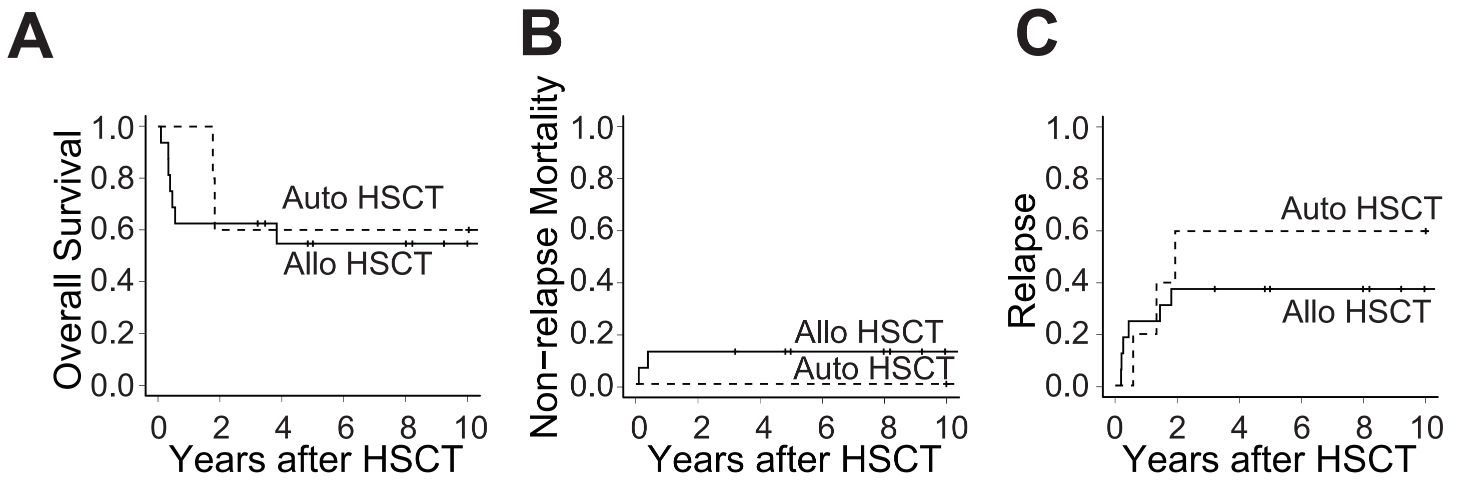

Supplemental Table

Supplemental Table 1. Details of induction and consolidation therapy

No.	Age/ Sex	Initial treatments	Response
1	17/M	JALSG ALL202-U protocol[1]	CR
2	44/F	JALSG ALL202-O protocol[2]	CR
3	30/M	HyperCVAD/MA, JALSG ALL97 induction therapy[3]	CR
4	53/M	HyperCVAD/MA, JALSG ALL97 induction therapy[3]	CR
5	23/M	HyperCVAD/MA, JALSG ALL97 induction therapy[3]	CR
6	36/M	HyperCVAD/MA, JALSG ALL202-O induction therapy[2]	CR
7	35/M	HyperCVAD/MA, CALGB 8811 induction therapy[4]	CR
8	19/M	HyperCVAD/MA	CR
9	20/F	HyperCVAD/MA	CR
10	35/F	HyperCVAD/MA	CR
11	47/M	HyperCVAD/MA	CR
12	34/M	HyperCVAD/MA	CR
13	23/F	HyperCVAD	CR
14	37/F	1 cycle of CHOP→JALSG ALL202-U protocol[1]	CR
15	18/M	HyperCVAD/MA, CHOP(2nd cycle),<br>JALSG ALL202-U consolidation therapy[1], DCVP(6th cycle)[5]	PR
16	34/M	HyperCVAD/MA, JALSG ALL202-O induction therapy[2]	PR
17	44/F	1 cycle of CHOP→HyperCVAD/MA	PR
18	31/M	HyperCVAD/MA	PR
19	26/M	1 cycle of CHOP→HyperCVAD/MA	PR
20	32/M	HyperCVAD/MA, CHOP(2nd cycle), ETP+PSL(4th cycle)	SD/PD
21	54/F	1 cycle of CHOP→1 cycle of ICE<br>→HD-MA, CALGB 8811 induction therapy[4]	SD/PD

Detailed chemo regimens; [1]JALSG ALL202-U; Sakura T et al., Blood 120:1464-1464, 2012; [2]JALSG ALL202-O; Sakura T et al., Leukemia 32:626-32, 2018; [3]JALSG ALL97: Jinnai I et al., Int J Hematol. 92:490-502, 2010; [4]CALGB 8811: Larson RA et al., Blood 85;2025-2037, 1995; [5]DCVP: Murata M et al., Rinsho Ketsueki 34:307-312, 1993.

Abbreviations: M, male; F, female; JALSG, Japan Adult Leukemia Study Group; HyperCVAD/MA, fractionated cyclophosphamide, vincristine, doxorubicin, and dexamethasone, and alternating with high-dose methotrexate and cytarabine; CALGB, Cancer and Leukemia Group B; CHOP, cyclophosphamide, doxorubicin, vincristine and prednisolone; DCVP, daunorubicin, cytarabine,

vincristine and prednisolone; ETP+PSL, etoposide and prednisolone; HD-MA, high-dose methotrexate and cytarabine; and ICE, ifosfamide, carboplatin and etoposide. Other abbreviations are shown in Tables 1 and 2.

Supplemental Table 2. Details of conditioning regimens for HSCT

Conditioning regimens	Standard-disease (N = 11)	Advanced-disease (N = 10)
Allo-HSCT		
MAC		
CY/TBI-based	7 (87.5%)	3 (37.5%)
MEL/TBI	0 (0.0%)	2 (25.0%)
BU/CY	1 (12.5%)	1 (12.5%)
RIC		
FLU/MEL/TBI-based	0 (0.0%)	2 (25.0%)
Auto-HSCT		
MAC		
CY/TBI-based	2 (66.7%)	0 (0.0%)
RIC		
MEAM/MEAM-like	1 (33.3%)	2 (100.0%)

Abbreviations are shown in Tables 1 and 2.

vincristine and prednisolone; ETP+PSL, etoposide and prednisolone; HD-MA, high-dose methotrexate and cytarabine; and ICE, ifosfamide, carboplatin and etoposide. Other abbreviations are shown in Tables 1 and 2.
